# Supplementary material for: Phylogenetic History and Phylogeographic Patterns of the European Wildcat (Felis silvestris) Populations
Source: Animals (Basel). 2023 Mar 6;13(5):953. doi: 10.3390/ani13050953 (PMC10000227; doi:10.3390/ani13050953)
Supplement: Supplementary file 1 [file animals-13-00953-s001.zip › supplementary/Velli_et_al._Supplementary_Material_02_EV.docx]

**SUPPLEMENTARY MATERIAL**

**Phylogenetic history and phylogeographic patterns of the European wildcat (*Felis silvestris*) populations**

Edoardo Velli ^1^*^†^, Romolo Caniglia ^1†^ and Federica Mattucci ^1†^

^1^ Unit for Conservation Genetics (BIO-CGE), Italian Institute for Environmental Protection and Research (ISPRA), Via Cà Fornacetta 9, 40064 Ozzano dell’Emilia, Italy

^†^ These authors contributed equally to this work

* Correspondence: [edoardo.velli@isprambiente.it](mailto:edoardo.velli@isprambiente.it)

**Results from Phylogenetic and Phylogeographic Analyses**

In this supplementary section, we reported all the details about the results of the phylogenetic and phylogeographic analyses performed using the 29 identified haplotypes obtained from the 715 sequences of wildcat, domestic and putative admixed samples. Since the three different phylogenetic trees constructed through Neighbour-joining, Maximum-likelihood and Bayesian phylogenetic approaches showed very concordant topologies for the main clades, we described in detail directly the topology of the tree generated by the Bayesian method implemented in Beast, which presented posterior probabilities of the main internodes > 0.90 (see Figure 2 in the Results section in the main text). The Bayesian phylogenetic tree clearly highlighted two main lineages (D and W) including five clades (I-III deriving from lineage D and IV-V from lineage W) and two sub-clades (Ia and Ib). Haplotypes were classified into three main categories according to the previous 31-STR Bayesian assignment tests performed by Mattucci et al. (2016): i) category “*d”* including haplotypes found only among domestic cats; ii) category “*dw*” including haplotypes found either in domestic, wild or putative admixed individuals and iii) category *“w”* including haplotypes found only among wildcats and putative admixed individuals. The subsequent phylogeographic analyses were conducted through network reconstructions on a reduced dataset pruned by the samples assigned to the domestic cat population, according to the previous STR analyses performed by Mattucci et al. (2016). Thus, in such network reconstructions (Figure 2c in the main text) lineage D was hence called lineage DW and the details reported here were referred only to the “*dw”* and “*w”* haplotypes.

Clade I included nine haplotypes shared by 312 individuals (140 wildcats, 125 domestic cats and 47 putative admixed individuals). It included four *dw* haplotypes (*dw*2, *dw*3, *dw*4, *dw*6), shared among 187 individuals, and was clearly separated into two sub-clades. Sub-clade Ia included haplotype *dw*4, which was the most frequent in the haplogroup DW, found in 146 individuals, 26.7% of them sampled in *Central Europe*, 25.3% in *Italy*, 25.3% in the *Balkans*, 21.2% in *Eastern Europe* and 1.4% in the *Iberian Peninsula*, and haplotype *dw*6, which was found in eight wildcats sampled in the *Balkans* and in one wildcat sampled in *Eastern Europe*. Sub-clade Ib included haplotype *dw*2, which was found in seven putative admixed individuals sampled in the *Scotland* population, and haplotype *dw*3, which was found in 15 putative admixed individuals and two wildcats sampled in *Eastern Europe* (60%), in six wildcat individuals sampled in the *Iberian Peninsula* (24%), one wildcat sampled in *Italy* (4%) and one wildcat sampled in the *Balkans* (4%).

Clade II included only two haplotypes and three individuals (one wildcat and two domestic cats) with haplotype *dw*7 found in a single wildcat sampled in Italy.

Clade III represented a basal group of lineage D counting four haplotypes including 133 individuals (33 wildcats, 86 domestic cats, and 14 putative admixed individuals). It included haplotypes *dw*1 and *dw*5, shared among 33 wildcats and 14 admixed individuals. Haplotype *dw*1 was found in 15 wildcats sampled in *Italy* (50%), in 12 putative admixed cats, in two wildcats sampled in *Eastern Europe* (46.6%), and in one single wildcat from the *Balkans* (3.3%). Haplotype *dw*5 was found in 14 wildcats sampled in the *Balkans*, in two putative admixed individuals sampled in *Eastern Europe* and in one wildcat sampled in *Central Europe* (Figure 3).

Clade IV included nine haplotypes found in 85 individuals, mainly from *Italy* (n = 52) and the *Iberian Peninsula* (n = 16) macro-regions (Supplementary Table S3). The most frequent haplotype of this clade was *w1*, detected in 62 (23.3%) individuals, 79.4% of which were sampled in *Italy*, 9.6% in the *Iberian Peninsula*, 6.3% in *Eastern Europe*, 3.2% in *Central Europe* and 1.6% in the *Balkans*. Haplotype *w*8 was found in nine individuals from the *Iberian Peninsula*, while haplotype *w*3 in seven putative admixed individuals from *Scotland* and one European wildcat from *Central Europe*. The remaining six haplotypes within clade IV were all found at low frequencies (Supplementary Table S3).

Clade V included five haplotypes, shared by 182 individuals mainly from *Central Europe* (n = 113) and *Balkans* (n = 42) macro-regions. The most frequent haplotype of this clade was *w4*, detected in 124 (45.9%) individuals 47.9% of which were sampled in *Central Europe*; 33.8% in the *Balkans*; 17.7% in the *Iberian Peninsula* and 0.8% in *Eastern Europe* (Supplementary Table S3). Haplotype *w*2 was found in 24 individuals (92.3%) sampled in *Central Europe* and in two individuals (7.7%) sampled in *Eastern Europe*, haplotypes *w*6 and *w*7 were found, respectively, only in 16 and 14 individuals from *Central Europe* whereas haplotype *w*9 was detected only in two individuals from the *Iberian Peninsula* (Supplementary Table S3).

**References**

Mattucci, F.; Oliveira, R.; Lyons, L.A.; Alves, P.C.; Randi, E. European Wildcat Populations Are Subdivided into Five Main Biogeographic Groups: Consequences of Pleistocene Climate Changes or Recent Anthropogenic Fragmentation? Ecol. Evol. 2016, 6, 3–22, doi:10.1002/ece3.1815

**Supplementary Figures**


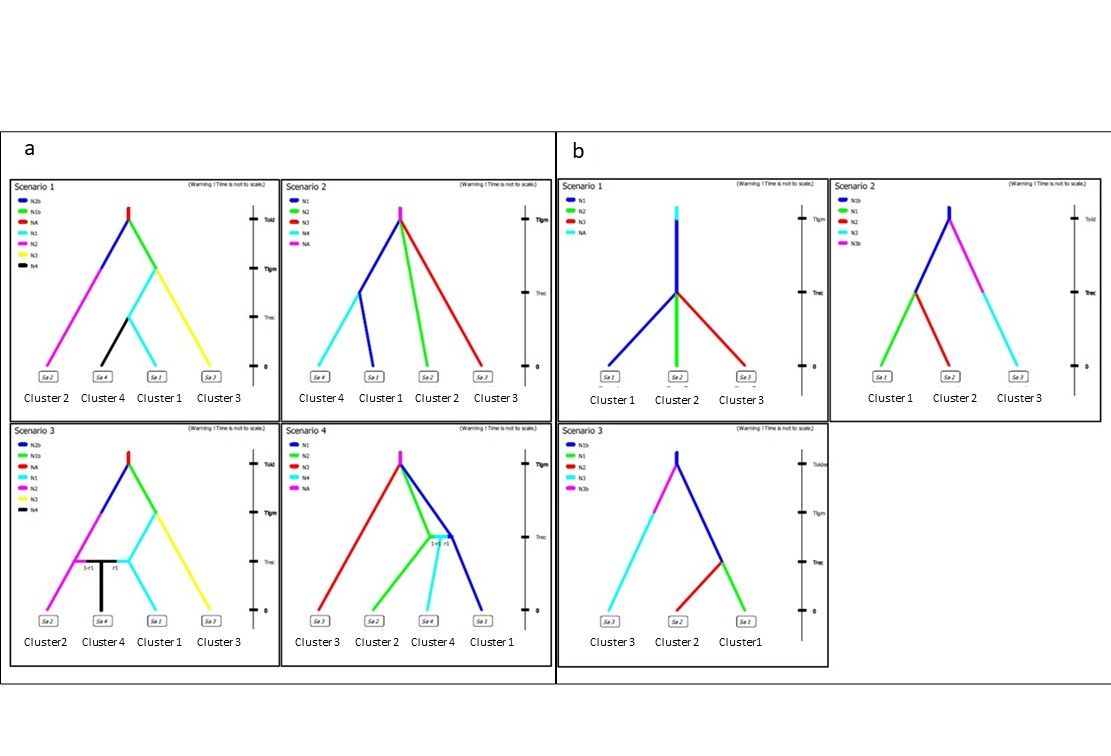


**Supplementary Figure S1:** **ABC simulated evolutionary scenarios**. Graphical representation of the different hypothesized scenarios with population sizes and divergence times inferred by ABC simulations, using a generation time g = 2 years. a) Scenarios hypothesized using lineage W haplotypes; b) scenarios hypothesized on lineage DW (lineage D pruned by domestic cats) haplotypes. Cluster numbers refer to the best *K* repartition obtained by SAMOVA analyses for each of the two lineages.


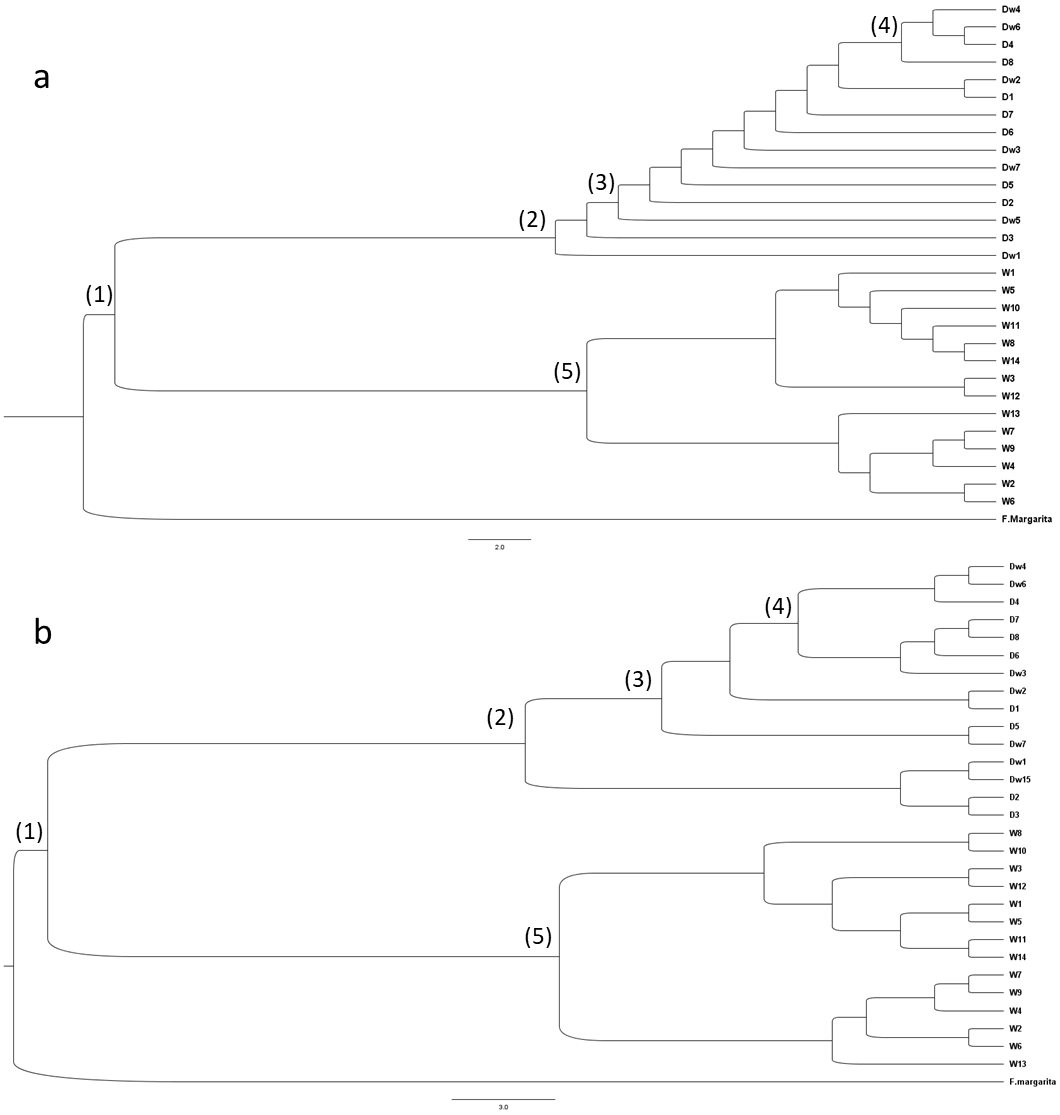


**Supplementary Figure S2: Neighbor-joining and maximum-likelihood topology trees.** a) Neighbour-joining tree and b) maximum-likelihood tree topologies showing phylogenetic relationships among ND5 haplotypes obtained from all the 715 analyzed cat samples. Numbers in brackets indicate well-supported nodes detected also in the Bayesian tree topology described in Figure 2.
